# A novel chalcone derivative suppresses melanoma cell growth through targeting Fyn/Stat3 pathway

**DOI:** 10.1186/s12935-020-01336-2

**Published:** 2020-06-18

**Authors:** Ling Tang, Jing Long, Keke Li, Xu Zhang, Xiang Chen, Cong Peng

**Affiliations:** 1 Department of Clinical Pharmacology, Xiangya Hospital, Central South University, Changsha, Hunan China; 2 Department of Dermatology, Xiangya Hospital, Central South University, Changsha, 410000 Hunan China; 3 Hunan Key Laboratory of Skin Cancer and Psoriasis, Xiangya Hospital, Central South University, Changsha, Hunan China; 4 Hunan Engineering Research Center of Skin Health and Disease, Xiangya Hospital, Central South University, Changsha, Hunan China

**Keywords:** Melanoma, Chalcone derivative, Fyn, Stat3, Cell growth

## Abstract

**Background:**

Fyn has been documented to have oncogenic features in multiple tumors, which might be a potential therapeutic target, however, few studies on the function role of Fyn and its specific inhibitors in melanoma.

**Methods:**

We investigated the impacts of Fyn and its inhibitor Lj-1-60 on melanoma through bioinformatics analysis, western blot, cell viability, cell cycle and apoptosis and xenograft tumor model as well as immunohistochemical staining. Pull-down and in vitro kinase assay were used to demonstrate Lj-1-60 targeting Fyn. Transcriptome sequencing and RT-PCR were adopted to confirm the potential mechanisms of Lj-1-60 in melanoma.

**Results:**

Our findings showed that Fyn was overexpressed in melanoma cells and knocked down of Fyn suppressed the proliferation of melanoma cells. To identify the potential inhibitors of Fyn, our in-house library including total of 111,277 chemicals was conducted to vitro screening, among those compounds, 83 inhibitors were further detected to explore the effect on melanoma cells growth and discovered a novel chalcone derivative Lj-1-60 that exhibited low cellular toxicity and high anti-tumor efficacy. Lj-1-60 directly was associated with Fyn and inhibited the Fyn kinase activity with Stat3 as substrate. What’s more, Lj-1-60 suppressed the proliferation of melanoma in vitro and in vivo through inducing cell cycle arrest and apoptosis. Moreover, the activation of Stat3 had also been abrogated both in Lj-1-60 treated melanoma cells or Fyn knocked down cells.

**Conclusion:**

Our study revealed a novel Fyn inhibitor that could significantly suppress melanoma growth, which is a promising potential inhibitor for melanoma treatment.

## Background

Cutaneous melanoma is a fatal skin cancer whose worldwide incidence has sharply increased in recent years. The pathogenesis of melanoma is known to have high complexity and diversity [1]. UV exposure has been proven to be a main cause linked to melanoma. An increasing body of evidence indicate that UV radiation induce a variety of mutations in genes, such as BRAF, RAS, C-Kit, NF1 and it enhances the activation of inflammation in melanoma [2]. Previously, clinical treatment for advanced metastatic melanoma was confined to dacarbazine and interleukin-2, and such a little benefit was achieved in a small proportion of patients with either therapy in the early 2000s [3]. More effective treatments have been developed including targeted therapy and immunotherapy with programmed death 1 (PD-1) and cytotoxic T-lymphocyte-associated protein 4 (CTLA-4). In contrast to traditional chemotherapy, targeting mutated BRAF inhibitors such as dabrafenib and vemurafenib, MEK inhibitors such as trametinib and cobimetinib have demonstrated remarkable improvement in overall survival and progression-free survival [4–6]. Treatment with immune checkpoint inhibitors including anti-CTLA4 and anti-PD-1 have been confirmed bringing extensive benefit for metastatic melanoma patients [7, 8]. However, there are some obvious limitations in targeted therapy and immunotherapy. Patients receiving BRAFi monotherapy or combination therapy with BRAFi and MEKi within 11 to 15 months resulted in drug resistance [9]. The efficacy of immunotherapy is approximately 20–30% even in melanoma [7]; therefore, It is essential that novel inhibitors are needed for melanoma treatment.

Fyn is an SRC family kinase (SFK) that contains 11 members. As a proto-oncogene, Fyn has been reported to have diverse biological functions such as cell growth, survival, adhesion and platelet activation [10]. Lewis-Tuffin et al. [11] observed that knocking down Fyn expression reduces glioma cells growth and migration through inhibiting activation of crucial genes such as catenin beta 1 (CTNNB1) and vav guanine nucleotide exchange factor 2 (VAV2). In a cholangio induced carcinoma model, deletion of Fyn repressed carcinoma cell migration and invasion through regulating the AMPK/mTOR signaling pathway [12]. Xi et al. [13] reported that Src family kinases including Fyn mediated the activation of the Stat family containing Stat3, and Src-specific inhibitors decreased the activation of Stat3 and reduced the growth of tumor cell in squamous cell carcinoma. Additional evidence showed that Fyn is one of most significantly altered genes in liver metastatic uveal melanoma, when compared with non-metastatic uveal melanoma [14]. In addition, Fyn has been identified as a melanoma biomarker through integrating human signaling networks with microarrays data [15], however, the details of Fyn in melanoma remain elusive.

In this study, we investigated the role of Fyn in melanoma, and screened out the potential inhibitors of Fyn as well as explored the possible molecular mechanisms of the candidate inhibitor in melanoma in vivo and in vitro, thus providing insight into the mechanism of melanoma and developing the anti-tumor drugs.

## Methods

### Chemicals and Lj-1-60 synthesis

Lj-1-60 ((*E*)-3-(4-bromo-3,5-dimethoxyphenyl)-1-(3-hydroxyphenyl) prop-2-en-1-one was synthesized as follows: a flask containing a stirred solution of freshly prepared sodium methylate (NaOMe) (CAS: 124-41-4, Aladdin, China) in methanol (MeOH) (CAS: 67-56-1, Aladdin, China) (1.0 M, 8 mL) was put at 0 °C, a solution of 3-hydroxyacetophenone (278 mg, 2 mmol) and 4-bromo-3,5-dimethoxybenzaldehyde (500 mg, 2 mmol) was added successively. The resulting solution was agitated at room temperature for 48 h, of which the solvent was then removed in vacuo and the remaining was carefully dissolved in deionized water (6 mL). The aqueous layer (pH 12) was washed with ether (Et_2_O) (CAS: 60-29-7, Aladdin, China) (3 × 2 mL), and controlled with the addition of concentrated hydrochloric acid (HCl) (CAS: 7647-01-0, Aladdin, China) until it reached pH 1. The aqueous layer was extracted with ethylacetate (EtOAc) (CAS: 141-78-6, Aladdin, China) (3 × 20 mL), and the mixture product were then concentrated. The produced yellow solid was recrystallized from ethanol–water to generate the product (*E*)-3-(4-bromo-3,5-dimethoxyphenyl)-1-(3-hydroxyphenyl) prop-2-en-1-one (427 mg, 68%). ^1^H NMR (1290UPLC-6540-Q-TOF, Agilent, America) (500 MHz, DMSO) δ results were as follows: 9.72 (s, 1H), 7.85 (d, *J* = 15.6 Hz, 1H), 7.59 (d, *J* = 15.6 Hz, 1H), 7.54 (d, *J* = 7.6 Hz, 1H), 7.36 (s, 1H), 7.28 (t, *J* = 7.8 Hz, 1H), 7.15 (s, 2H), 6.96 (d, *J* = 8.0 Hz, 1H), 3.81 (s, 6H); ^13^C NMR (125 MHz, DMSO) δ 189.7, 158.2, 157.3, 144.0, 139.4, 135.8, 130.3, 123.6, 120.8, 120.2, 115.2, 105.9, 102.8, 57.2; ESI-HRMS calcd for C_17_H_16_BRO_4_ [(M+H)^+^] 363.0226, 365.0206, and found 363.0225, 365.0210. 50 mM Lj-1-60 was prepared as stock and DMSO as solvent, the stock of Lj-1-60 was then diluted to 5 mM, and working solution was further diluted with DMSO stored at − 20 °C for further research.

### Cell lines and culture

Human melanoma cell lines (Sk-Mel-5, Sk-Mel-28 and A375), human keratinocyte cell (HaCaT), rat embryonic ventricular cardiomyocytes (H9C2) and mouse skin epidermal cell line (JB6), human embryonic kidney cells (HEK293T) were purchased from the American Type Culture Collection (ATCC), all of which were cultured in Dulbecco’s modified Eagle’s medium (BI, Israel) containing 10% fetal bovine serum (FBS) (BI, Israel) and 1% penicillin–streptomycin at a temperature of 37 °C with 5% CO_2_. Melanocyte cells (PIG1) was obtained from the Department of Dermatology, Third Xiangya Hospital (initially a gift originated from prof. Caroline who come from Le Poole Loyola University Chicago) were cultured with 254 medium (Gibco) containing 5% FBS and 1% HMGS (Gibco) 37 °C with 5% CO_2_. DMEM was used for above all cell lines except for PIG1 [16].

### Lentivirus infection

The virus packaging protocol was described as our lab performed previously [17]. In short, target gene plasmids (shMock, shFyn#1 and shFyn#4) plus with package plasmids including psPAX2 and pMD2G were transfected into HEK 293T cells for 48 to 72 h. The supernatant was collected and centrifuged at 3500 rpm for 20 min. The lentivirus was used to infect melanoma cells with the help of 10 μg/mL polybrene. Cells were then selected with 2 µg/mL puromycin until the control (uninfected) cells died.

### Apoptosis and cell cycle assay

For apoptosis assay, melanoma cells treated with 2 µM Lj-1-60 for 48 h and Fyn knocked down melanoma cells were stained with double reactive dyes (FITC-Annexin V and PI) following by manufacturer’s instructions (Beyotime, China). For cell cycle assay, pretreated cells were firstly fixed with ice cold 70% ethanol overnight, and then stained with propidium iodide (PI) (Beyotime, China), the mixture was incubated for 30 min at room temperature avoiding light. Cells were detected by flow cytometry (Becton, Dickinson Company, USA) and data analysis was carried out using FlowJo software. Above the detailed methods were referred to Nian Liu’s et al. protocal [18]. All samples were tested three times.

### Immunoblotting

The protocol was performed according the established method in our lab [18]. Cells were lysed in RIPA buffer (Beyotime, China) with cocktail and phosphatase inhibitors (Selleck, USA), and the concentration was tested by protein assay kit (Beyotime, China). Proteins were then separated by 10% SDS-PAGE and transferred to polyvinylidene fluoride membranes (Millipore, USA). Membranes carrying on proteins were incubated with primary antibody at 4 °C for overnight, next day, the membranes were incubated with secondary antibody at room temperature for an hour. Finally, they were imaged by chemiluminescence by way of ECL Regents (NCM Biotech, China). The primary antibodies were used as follows: PARP (CST, USA), Bcl-2 (Proteintech, China), BAX (Proteintech, China), P53 (CST, USA), p-P53 (CST, USA), P21 (CST, USA), P-Stat3 (CST, USA), and Stat3 (CST, USA).

### Molecular docking

Molecular modeling was performed using SYBYL-X 2.1.1 from Tripos Associates Inc. Our in-house compound library composed of 111,277 purchased and synthesized compounds was used for virtual screening to find novel inhibitors of Fyn which was described as previously [19]. Crystal structure of Fyn kinase domain complexed with staurosporine obtained from the Protein Data Bank (PDB ID:2DQ7) was employed as the docking template for structure-based virtual screening using multi-precision docking. Before docking, its preparation was conducted including by removing unbounded water and salts, adding hydrogen atom and retaining energy minimization. Ligands were docked into the active site of Fyn.

### Ex vivo pull-down assay

Lj-1-60-linked Sepharose 4B beads pretreated as described previously [20]. Proteins (approximately 500 μg) coupled with Lj-1-60-linked Sepharose 4B beads reacted in incubation buffer, and Sepharose 4B alone as control. Beads containing proteins were washed three times with washing buffer after rocking overnight at 4 °C mildly, and proteins bound to Lj-1-60-linked Sepharose 4B beads were subjected to western blotting.

### In vitro phosphorylation of stat3

Recombinant human Stat3 was purchased from CUSABIO. Stat3 was phosphorylated by 50 nM Fyn (EMD Millipore Corporation) in kinase buffer with 1 mM adenosine triphosphate (ATP) at 30 °C for 40 min [21]. For the “unphosphorylated” sample, Fyn was incubated in same buffer that lacked Stat3 as the substrate. For the “treatment group” samples, various concentrations of Lj-1-60 were added to this system. After incubation, the samples were detected by western blotting. The total and phosphorylated Stat3 levels were visualized by chemiluminescence ECL reagents.

### Cell viability

Melanoma cells and non-tumor cells (2 × 10^3^ cells) selected in our study were seeded into 96-well plates per well, and complete medium containing various concentrations of Lj-1-60 was added into the plates, which was then followed by culturing for 24, 48, and 72 h, and DMSO as a control. For the concentration of DMSO, we choose the concentration in response to the highest concentration of Lj-1-60 as control. Cell viability was detected with cell counting kit-8 (CCK-8 kit) (Selleck, USA) by measuring the OD value at the wavelength of 450 nm [18]. Each experiment performed at least three times independently and each group had four replicates. GraphPad Prism 6.0 was used to calculate the half-maximal inhibitory concentration (IC50) value within this step.

### Colony formation assay

Cells (1.5 × 10^3^ cells/well) were treated with 2 µM Lj-1-60 for 24 h with DMSO as control, and the medium containing Lj-1-60 was removed after 24 h of culture and was replaced with complete medium every 4 days for 2 weeks. Colonies fixed with 4% paraformaldehyde were stained with 0.5% crystal violet (Beyotime, China), and counted with ImageJ software [16].

### Xenograft tumor model

A number of 1 × 10^6^ melanoma cells Sk-Mel-5 were injected subcutaneously in the flanks of nude mice (nu/nu). Once the size of the tumor reached up to 50 mm^3^, a total of 15 mice were randomly divided into three groups which were 20 mg/kg group, 40 mg/kg group and vehicle group, respectively, each dose group were treated with the corresponding dose of 20 or 40 mg/kg Lj-1-60 with intraperitoneal injection, and normal saline was used in vehicle group for once a day for 2–3 weeks. The weight of mice was monitored, and the size of tumor was gauged by vernier caliper every other day. The formula of tumor volume V = 1/2 (length × width^2^) [16].

### Immunohistochemical staining

Mice tumors tissues were fixed, embedded and sectioned, slides carrying on tissues were heated at 65 °C for 2 h, sections were then deparaffinized and rehydrated with gradient ethanol. Endogenous peroxidase was inhibited by treatment with 3% hydrogen peroxide at room temperature for 10 min after antigen retrieval. Subsequently, the slides incubated with primary antibody (Ki67, Abcam) overnight, and then were incubated with secondary antibody at room temperature for an hour. The slides were finally stained with DAB, counterstained with hematoxylin and mounted in neutral balsam [19].

### RNA-sequencing and quantitative real-time PCR analysis

After treatment with Lj-1-60 for 48 h, melanoma cells Sk-Mel-5 and Sk-Mel-28 were harvested for cDNA library construction, purification and sequencing, all procedures were completed at Wuhan Huada Sequencing Company. The exported primary results were analyzed by the Huada Gene Interactive Reporting System. RNA for further validation was extracted from melanoma cells pretreated with 2 µM Lj-1-60 for 24 h and 48 h, and RNA of Fyn knocked down melanoma cells was also extracted. The synthesized cDNA was used as templates for real-time PCR using SYBR Green qPCR mix (CWBiotech, China). The primers used in this protocol are listed in Additional file 1: Table S1.

### Statistical analysis

All data produced in our study are shown as the mean values ± S.D, and performed for at least three independent experiments. All data was normalized and the statistic differences were determined by Student’s t test, one-way and two-way ANOVA using GraphPad Prism 6.0. P < 0.05 was regarded as statistical difference.

## Results

### Fyn is overexpressed in melanoma cells and knockdown of Fyn induces apoptosis in melanoma cells

**Fig. 1:**
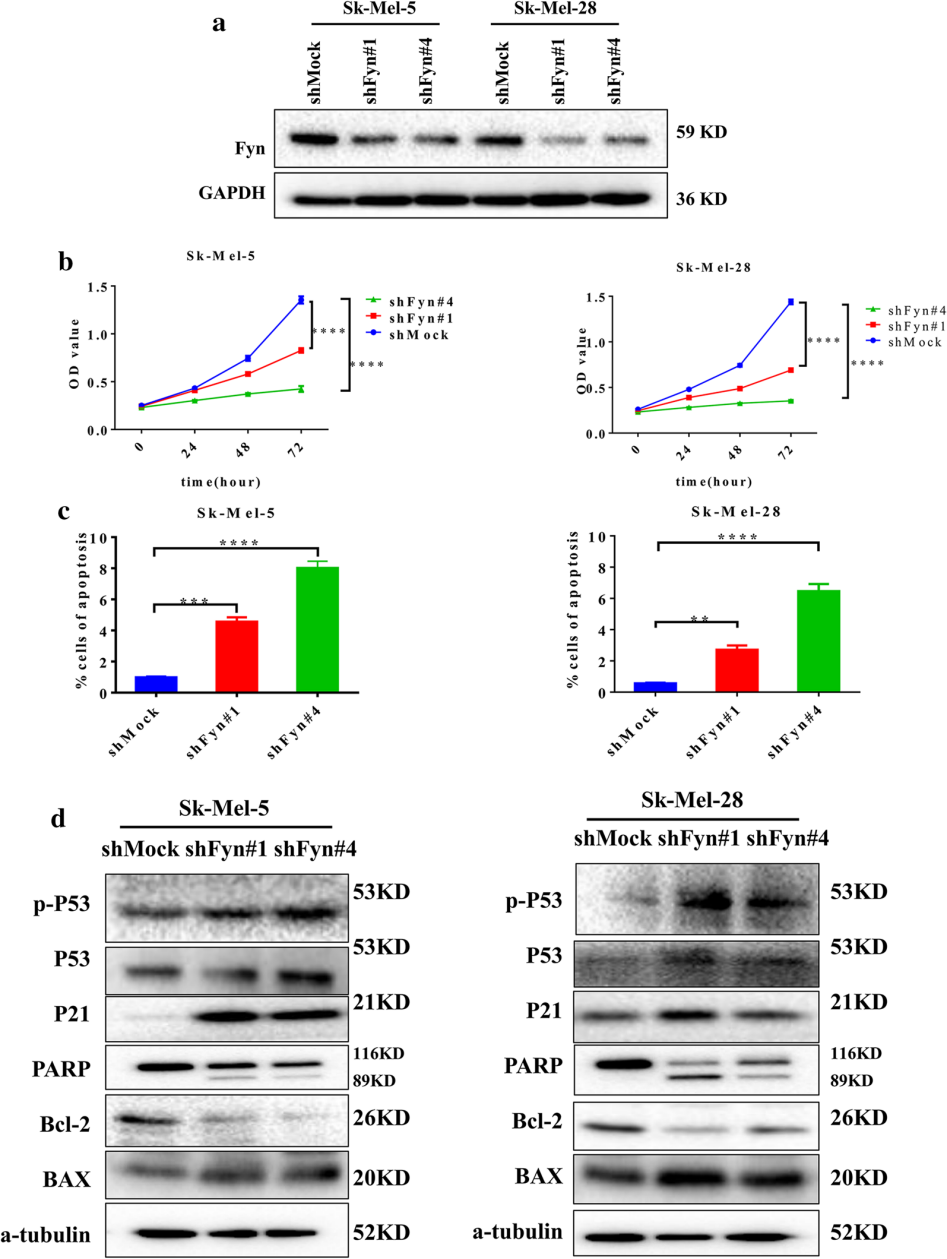
Fyn is overexpressed in melanoma cells and knockdown of Fyn induces apoptosis in melanoma cells. **a** Knocked down of Fyn with two independent shRNA in melanoma cells Sk-Mel-5 and Sk-Mel-28, the expression of Fyn was assessed by western blotting with anti-Fyn antibody. **b** Cell viability of knocked down of Fyn in melanoma cells Sk-Mel-5 and Sk-Mel-28 were tested by CCK-8 assay. All samples represented at least for four replicates with three independent experiments, ****P < 0.0001. **c** Apoptosis was determined by flow cytometry with Annexin V and PI double staining in Fyn knocked down melanoma cells Sk-Mel-5 and Sk-Mel-28, data was presented as mean (n = 3) ± SD, **P < 0.01, ***P < 0.001, ****P < 0.0001. **d** Knocked down of Fyn in melanoma cells Sk-Mel-5 and Sk-Mel-28, Cell lysates were quantified and separated by SDS-PAGE. Western blot of the indicated proteins was done as described in “Methods”. Specific antibodies were used for detection of indicated proteins and a-tubulin was used as an internal control

To verify the relationship between the expression of Fyn and melanoma, the relative expression of Fyn between melanoma tissues and normal tissues was evaluated based on GEO databases (GSE114445 and GSE29359). The results showed that Fyn is highly expressed in primary melanoma compared with control (Additional file 2: Fig S1A). To investigate the function role of Fyn in melanoma cells, we successfully generated Fyn knocked down melanoma cells (Fig. 1a). Our findings showed that knockdown of Fyn significantly inhibited melanoma cells proliferation, Specifically, compared with shMock group, the rate of proliferation in shFyn#1 group reduced by 38.9%, and in shFyn#4 group reduced by 68.7% at 72 h in melanoma cell Sk-Mel-5. In melanoma cell Sk-Mel-28, we obtained the similar results, the rate of proliferation in shFyn#1 group reduced by 51.9%, and in shFyn#4 group reduced by 75.5% at 72 h (Fig. 1b). We also observed that Fyn knocked down melanoma cells induced cellular apoptosis. As shown in Fig. 1c, the proportion of Annexin V-positive cells indicating the early and late apoptosis was increased 4.6 times in shFyn#1 group and 8.0 times in shFyn#4 group compared with shMock group in melanoma cell Sk-Mel-5 while 2.7 times in shFyn#1 group and 6.5 times in shFyn#4 group in melanoma cell Sk-Mel-28. We further examined the expression of cell cycle- and apoptosis-related genes in Fyn suppressed cells, and the results revealed that Fyn knockdown increased p-P53, P53, P21, and BAX expression and the cleavage of PARP, while it downregulated Bcl-2 (Fig. 1d), suggesting the crucial role of Fyn in melanoma cell apoptosis.

### Lj-1-60 is a novel inhibitor targeting Fyn protein kinase

**Fig. 2:**
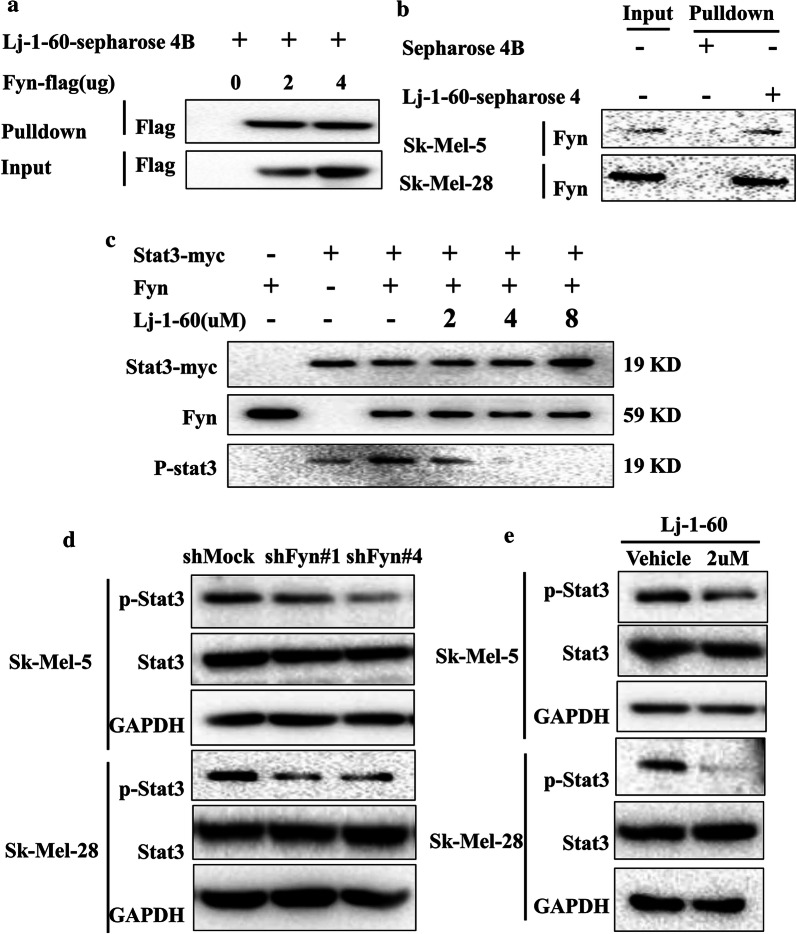
Lj-1-60 is a novel inhibitor targeting Fyn protein kinase. **a** HEK293T cells transfected with 2 μg and 4 μg of Fyn-flag plasmids for 36 h, it was then harvested and assessed by pull-down assay. Cell lysates incubated with Sepharose coupled with Lj-1-60 were subjected to SDS-PAGE, and analyzed by immunoblotting with antibody anti-flag. **b** Melanoma cell lysates for pull-down assay was detected by immunoblotting with anti-Fyn antibody. **c** In vitro kinase assay. A reaction mixture of substrate human recombinant protein Stat3 with myc-tag (676-770 aa), Fyn kinase and Lj-1-60 were incubated at 30 °C for 40 min and subjected to immunoblotting with indicated antibodies. Data are representative of three independent experiments. **d** Knocked down of Fyn in Sk-Mel-5 and Sk-Mel-28 cell lines by two independent shRNA. Total cell lysates were subjected to immunoblotting using indicated antibodies. Data are representative of three independent experiments. **e** Lj-1-60 treated in Sk-Mel-5 and Sk-Mel-28 cell lines for 48 h with indicated concentration and analyzed by immunoblot using antibodies p-Stat3(Tyr 705), Stat3, and GAPDH as loading control

Previous studies and our above results proved that Fyn might be a promising target for melanoma therapy. To identify specific inhibitors targeting Fyn, we performed virtual screening to find potential inhibitors from our in-house library that contains 111,277 compounds. Multi-precision docking screening was adopted to improve the efficiency of docking. Specifically, Surflex-Dock Screen mode with low accuracy was firstly used to dock hit compounds into the active binding site of Fyn, and the output top 500 compounds according to the total score were then analyzed under the mode with standard accuracy, the top 300 compounds produced from the previous step were further analyzed with more exhaustive accuracy. Finally, the top 83 hit compounds output from the last docking were validated in melanoma cells Sk-Mel-28 (Additional file 3: Fig S2A). Among those inhibitors, the chemical ((E)-3-(4-bromo-3,5-dimethoxyphenyl)-1-(3-hydroxyphenyl) prop-2-en-1-one) (Lj-1-60) (Additional file 3: Fig S2B) exhibited high antitumor activity. Lj-1-60 was discovered to properly dock to the ATP-binding site of Fyn. Concretely, the carbonyl group of Lj-1-60 acted as a hydrogen bonding acceptor which is interacted with the backbone amide group of Met85, while the hydroxyl group acted as a hydrogen bonding donor bound to the backbone carbonyl group of Met85. Meanwhile, the methoxyl group also serves as a hydrogen bonding acceptor interacting with Asp148 (Additional file 3: Fig S2C, D). The hydrophobic reaction of Lj-1-60 with Fyn contributes to the highly activation of the inhibitor for Fyn. Next, we examined whether Lj-1-60 directly bind to Fyn. Increasing amounts of the Fyn-Flag plasmids transfected into HEK293T cells for 36 h, cell lysates were incubated with Lj-1-60-Sepharose 4B beads for pull-down assays. The results showed that Lj-1-60 bound to Fyn in a dose-dependent manner (Fig. 2a). To further validate that Lj-1-60 targeting Fyn, melanoma cell lysates (Sk-Mel-5 and Sk-Mel-28) were incubated with Lj-1-60-Sepharose 4B beads. Our findings suggested that Fyn bound to the Lj-1-60-Sepharose 4B bead complex in melanoma cells (Fig. 2b). Given that Lj-1-60 is bound to Fyn, we hypothesized that this compound may affect Fyn protein kinase activity. Stat3 is a well-known substrate of Fyn and plays crucial role in melanoma tumorigenesis; thus, Stat3 acts as a substrate to test Fyn protein kinase activity. As shown in Fig. 2c, the phosphorylation of Stat3 dramatically decreased following in presence of Lj-1-60 with a dose-dependent manner, indicating that this compound could directly inhibit Fyn protein kinase activity by inhibiting the phosphorylation of Stat3. Then, we examined the effect of Lj-1-60 on Fyn induced the phosphorylation of Stat3 in vivo, as shown in Fig. 2d, e, both knockdown of Fyn and Lj-1-60 treatment in melanoma cells led to decrease level of phosphorylation of Stat3, indicating that Lj-1-60 is a novel inhibitor targeting Fyn.

### Lj-1-60 suppresses the proliferation of melanoma cells in vitro

**Fig. 3:**
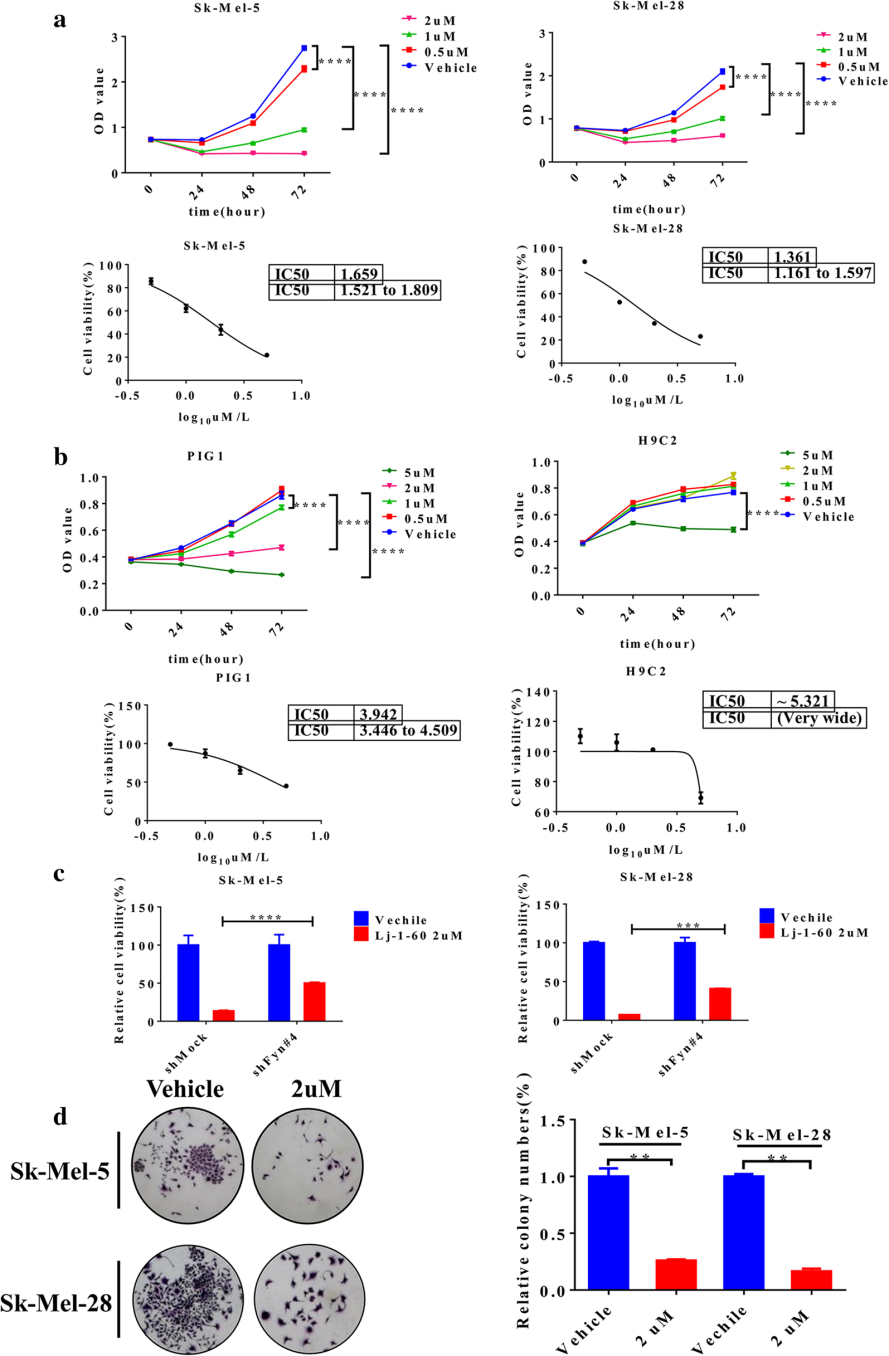
Lj-1-60 suppresses the proliferation of melanoma cells in vitro. **a** Melanoma cells Sk-Mel-5 and Sk-Mel-28 seeded into 96-well plates were treated with various concentrations of Lj-1-60 for 0, 24, 48, 72 h, cell viability was tested by CCK-8 kit, the value of IC50 was calculated by Graphpad prism 6. All sample represented at least for three replicates with three independent experiments, ****P < 0.0001 by two-way ANOVA. **b** Cell viability in non-tumor cells including PIG1 and H9C2 was measured as described in “Methods”. Data are Mean ± SD (n = 4 wells), ****P < 0.0001 by two-way ANOVA. **c** Inhibition of Fyn and treated with 2 μM Lj-1-60 for 48 h in melanoma cells. Cell viability was examined by CCK-8 assay, ***P < 0.001, ****P < 0.0001. **d** Melanoma cells were seeded into 6-well plate with complete medium overnight, cells were replaced with medium containing 2 μM Lj-1-60 for 24 h, and complete medium cultured for another 2 weeks, the number of clones was stained with crystal violet and counted with software image J. The data represented here are expressed as the mean(n = 3) ± S.D. **P < 0.01

Next, we tested the anti-proliferative effect of Lj-1-60 on various melanoma cells. Melanoma cells (Sk-Mel-5, Sk-Mel-28) were treated with various concentrations of Lj-1-60 (0.5 μM, 1 μM, and 2 μM), and our findings indicated that Lj-1-60 markedly reduced cell viability in a time- and dose-dependent manner, with IC50 values of 1.65 µM (Sk-Mel-5) and 1.36 µM (Sk-Mel-28) respectively (Fig. 3a). The anti-proliferative effect of Lj-1-60 was also demonstrated in another melanoma cell line (A375) (Additional file 4: Fig S3A). Meanwhile, there was less cytotoxicity observed in non-tumor cells, such as PIG1 and H9C2 cells. The IC50 value of Lj-1-60 in melanocyte cells PIG1 was 3.9 µM, while the IC50 value in H9C2 cells was over 5.3 µM (Fig. 3b). Similar results were observed in other non-tumor cell lines (HaCaT and JB6) with IC50 values of 3.3 µM and 4.6 µM, respectively (Additional file 4: Fig S3B, C). To further verify the specificity of Lj-1-60 targeting Fyn, we explored the effect of this drug on cell viability of Fyn knocked down melanoma cells and the result exhibited that anti-tumor effect of Lj-1-60 was partially reduced in Fyn knocked down cells (Fig. 3c), indicating that Lj-1-60 suppresses the growth of melanoma cells at least partly by targeting Fyn. Moreover, as shown in Fig. 3d, Lj-1-60 significantly decreased the ability of colony formation in both cell lines compared with that of the vehicle group, suggesting that Lj-1-60 suppresses melanoma cells growth in vitro.

### Lj-1-60 induces melanoma cell apoptosis and cell cycle arrest in G2/M phase

**Fig. 4:**
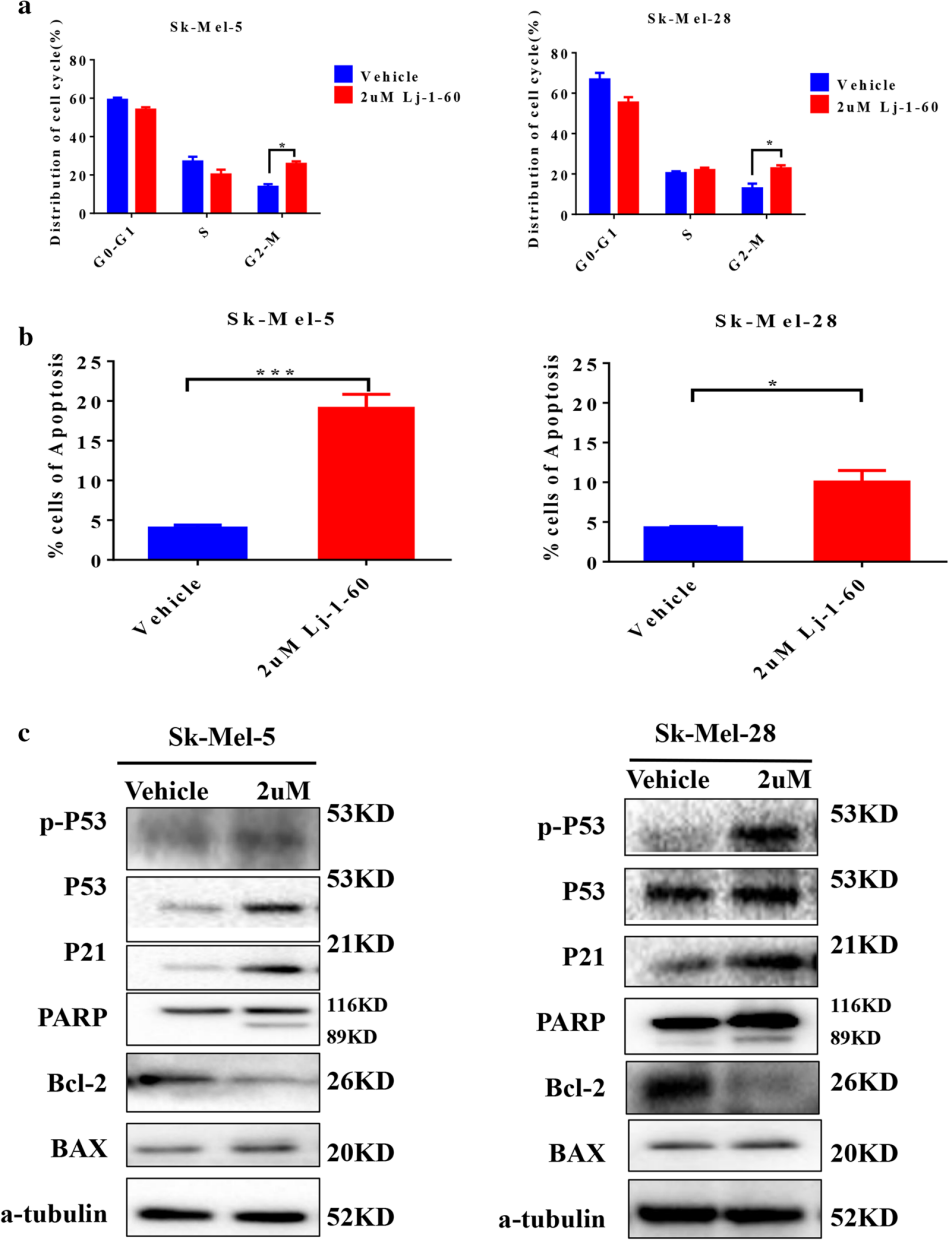
Lj-1-60 induces melanoma cell apoptosis and cell cycle arrest in G2/M phase. **a** Cell cycle distribution was detected by flow cytometry treated with 2 μM Lj-1-60 for 48 h in melanoma cells Sk-Mel-5 and Sk-Mel-28, and DMSO as vehicle according to the manufacture’s instruction. The results represented the mean (n = 3) ± SD of each group, *P < 0.05. **b** Melanoma cells Sk-Mel-5 and Sk-Mel-28 treated with 2 μM Lj-1-60 for 48 h, and the percentage of apoptosis was examined by flow cytometry as described in the "Methods" with Annexin V and PI double staining. Data were represented as mean (n = 3) ± SD, *P < 0.05, ***P < 0.001. **c** Melanoma cells Sk-Mel-5 and Sk-Mel-28 treated with 2 μM Lj-1-60 for 48 h, Total cell lysates were quantified and subjected to western blotting detected with the indicated antibodies

Given that Lj-1-60 suppresses melanoma cell growth, we continued to explore the effect of Lj-1-60 on cell cycle and apoptosis. Regarding cell cycle progression in melanoma cells, Lj-1-60 significantly increased the number of melanoma cells (Sk-Mel-5, Sk-Mel-28) at G2/M phase, by 46.3% and 43.6%, respectively (Fig. 4a). As with apoptosis, melanoma cells were treated with 2 μM Lj-1-60 for 48 h, as shown in Fig. 4b, the number of early and late apoptotic cell populations in the two cell lines (Sk-Mel-5, Sk-Mel-28) increased by 19.25% and 10.2%, respectively. In addition, melanoma cells treated with 2 μM Lj-1-60 for 48 h were further detected for changes in the expression of cell cycle- and apoptosis pathway-related proteins with western blotting. After Lj-1-60 treatment, the expression of p-P53, P53, P21, and Bax and the cleavage of PARP was raised, while Bcl-2 had been down-regulated (Fig. 4c), suggesting that Lj-1-60 induced melanoma cell cycle arrested in G2/M phase and apoptosis.

### Lj-1-60 decreases the growth of tumor xenografts in nude mice

**Fig. 5:**
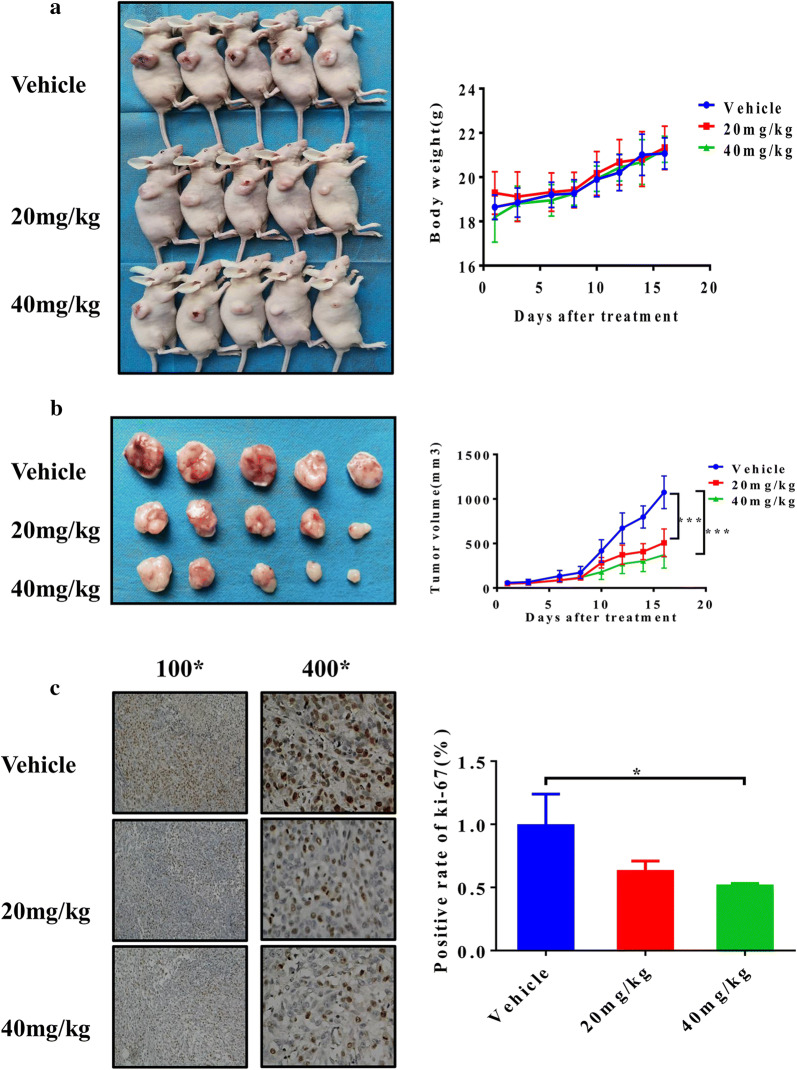
Lj-1-60 decreases the growth of tumor xenografts in nude mice. **a** Melanoma cells Sk-Mel-5 (1 × 10^6^ cells) were grafted into nude mice. When tumors reached up to approximately 50 mm^3^, the tumor-bearing mice were randomized for intraperitoneal injection of 20 or 40 mg/kg of Lj-1-60 once a day for 2–3 weeks as described in “Methods”. **b** The tumor growth and body weight were measured twice per day. The results are shown as the mean tumor volume ± SD, and an asterisk (***) indicates a significant difference (P < 0.001). **c** Immunohistochemical staining of ki-67 (a marker of cell proliferation) in the tumor tissues of xenograft nude mice. Data are represented as means (n ≥ 3) ± SD. *P < 0.05

To further demonstrate the function role of Lj-1-60 in melanoma in vivo, we conducted a xenograft study in nude mice. Two doses of Lj-1-60 (20 mg/kg, 40 mg/kg) prepared with saline was administered via intraperitoneal injection once a day for 2–3 weeks. As shown in Fig. 5, both low and high doses of Lj-1-60 inhibited tumor cell growth in vivo, but no significant statistical difference in body weight of mice was observed in the treatment groups when compared with the vehicle group (Fig. 5a). The tumor volume was reduced by 52.8% in 20 mg/kg Lj-1-60 treated group and 65.4% in 40 mg/kg Lj-1-60 treated group compared with vehicle group (Fig. 5b). Immunohistochemical staining also showed that Ki-67 (a tumor proliferation marker) was significantly reduced in the Lj-1-60-treated mice especially in 40 mg/kg Lj-1-60 treated group in comparison to that of the vehicle group (Fig. 5c), suggesting that Lj-1-60 suppresses melanoma growth in vivo.

### Analysis of gene expression profiles involved in melanoma cells altered by Lj-1-60 treatment

**Fig. 6:**
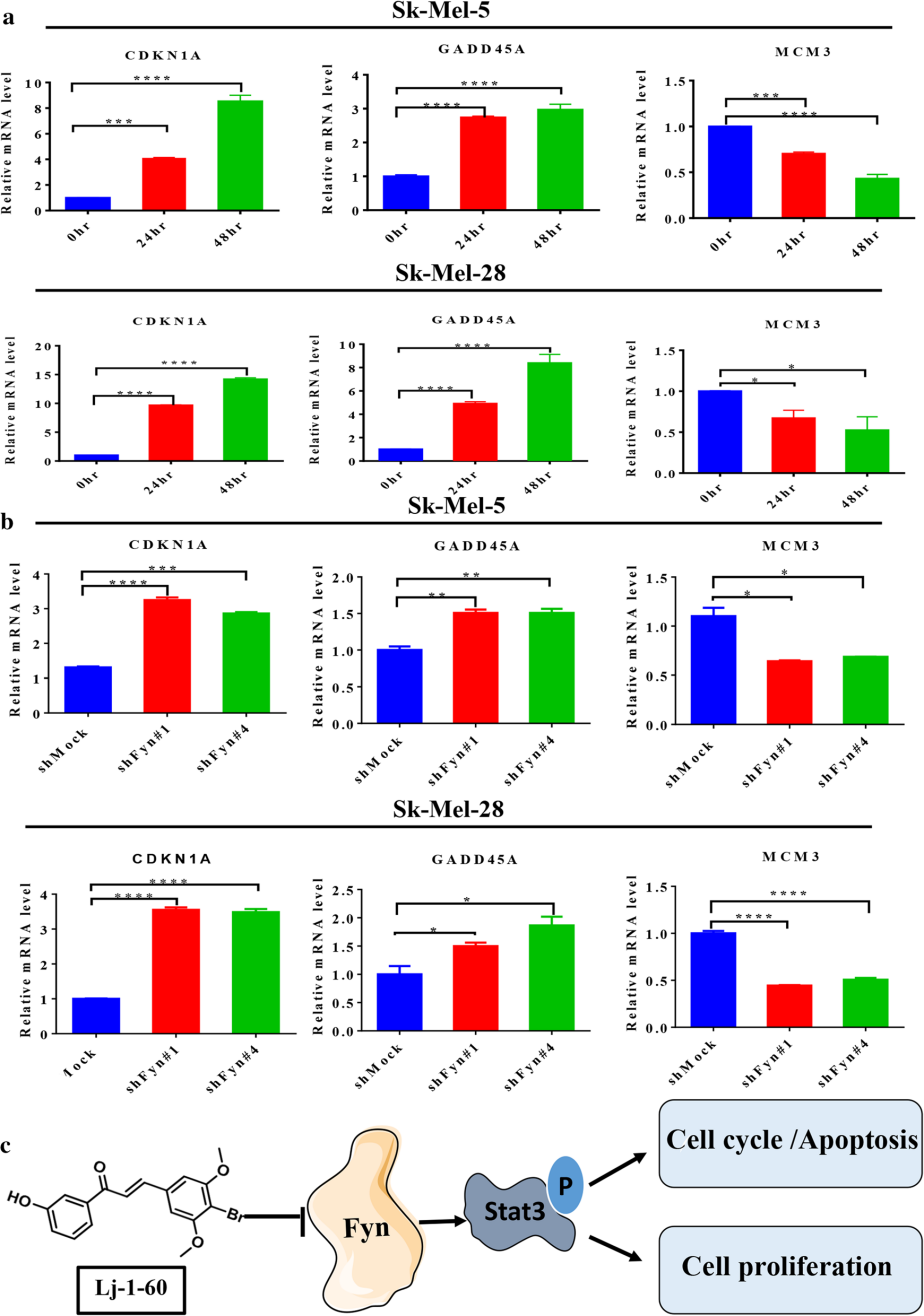
Analysis of gene expression profiles involved in melanoma cells altered by Lj-1-60. **a** The mRNA expression of CDKN1A, GADD45A, MCM3 in melanoma cells Sk-Mel-5 and Sk-Mel-28 was measured by RT-PCR. Total RNA was extracted from the cells treated with 2 μM Lj-1-60 for 24, 48 h. Data were expressed as mean (n = 3) ± SD, *P < 0.05, ***P < 0.001, ****P < 0.0001. **b** The mRNA expression of CDKN1A, GADD45A, MCM3 in melanoma cells Sk-Mel-5 and Sk-Mel-28 was examined by RT-PCR. Total RNA was extracted from cells knocked down of Fyn with two independent shRNA. Data were expressed as mean (n = 3) ± SD, *P < 0.05, ***P < 0.001, ****P < 0.0001. **c** Structural model. Lj-1-60 inhibits the proliferation of melanoma and induces cell cycle arrested in G2/M phase and apoptosis by targeting Fyn through inhibiting the phosphorylation of Stat3

To investigate the potential mechanism connecting with the effect of Lj-1-60 on melanoma cells, we examined the alteration of gene expression profiles from melanoma cells treated with Lj-1-60 for 48 h with RNA-seq. Hierarchical clustering of genes through quantitative and normalization demonstrated that the significantly differences induced by treatment of Lj-1-60 in the transcriptome. In total, 2757 genes were upregulated and 729 genes were downregulated in the Lj-1-60-treated group in the melanoma cell line Sk-Mel-5 (Additional file 5: Fig S4A). Approximately 4022 genes were upregulated and 837 genes were downregulated in the melanoma cell line Sk-Mel-28 (Additional file 5: Fig S4D). KEGG pathway analysis indicated that multiple signaling pathways were changed in Lj-1-60-treated melanoma cells; among those pathways, DNA replication, cell cycle and P53 signaling pathways were significantly enriched in Sk-Mel-5 cells (Additional file 5: Fig S4B) and Sk-Mel-28 cells (Additional file 5: Fig S4E). Gene set enrichment analysis (GSEA) further confirmed that Lj-1-60 treatment in melanoma cells led to alteration in numerous signaling pathways, especially for the cell cycle (Additional file 5: Fig S4C) and DNA replication (Additional file 5: Fig S4F). To validate the transcriptome results, we selected genes with significant expression differences related with the cell cycle and apoptosis for further validation using quantitative real-time PCR (RT-PCR). Consistent with the sequencing results, RT-PCR confirmed that GADD45A and CDKN1A were upregulated in the Lj-1-60-treated group, while minichromosome maintenance 3 (MCM3) was downregulated in melanoma cells Sk-Mel-5 and Sk-Mel-28 (Fig. 6a). Moreover, given Lj-1-60 is a potential inhibitor for Fyn, we also examined those genes expression in Fyn knocked down melanoma cells. In line with the results of Fig. 6a, we observed that GADD45A and CDKN1A were upregulated in the Fyn knocked down cells, but MCM3 was downregulated (Fig. 6b).

## Discussion

Melanoma is a malignant tumor whose etiology is unclear, and researches focus on the development of anti-tumor drugs were constantly going on due to the resistance and reoccurrence of target therapy and immune therapy. In this regard, we investigated the impact of Fyn on melanoma, and screened out the potential inhibitors of Fyn as well as explored the possible molecular mechanisms of the candidate inhibitor in melanoma. We found that knocked down of Fyn inhibited the proliferation and induced cell cycle arrest and apoptosis in melanoma Furthermore, we discovered that Lj-1-60 screened out from our in-house lab targeted Fyn and exhibited a strong anti-tumor effect in vivo and in vitro, which is helpful in providing insight into the mechanism of melanoma and developing the anti-tumor drugs.

Activation of Fyn has been observed in multiple tumors including melanoma, glioblastoma, squamous cell carcinoma and prostate cancer [22]. In our study, we compared the Fyn expression between melanoma tissues and normal tissues from RNA-seq results of GEO databases (GSE114445 and GSE29359) and also found that Fyn is highly expressed in melanoma, and knocked down of Fyn markedly inhibits melanoma proliferation and induces apoptosis (Fig. 1), indicating the crucial role of Fyn in melanoma. A previous study demonstrated that the activation of Fyn induced by the MKP-1 regulated signaling pathway facilitates cellular transformation [23]. Melanoma cells are hyperactive in the RAS-MAPK pathway and Fenton et al. revealed that the RAS activates Fyn leading to increased cell migration and invasion, while suppression of Fyn expression abrogates RAS induced malignant phenotype [24]. Given that Fyn phosphorylates and activates downstream molecules, Fyn has various biological functions in tumorigenesis such as migration and adhesion. Fyn was reported to mediate the phosphorylation of PIKE-A at site T172 that inhibits AMPK signaling pathway, and results in blocking glioblastoma cell suppression activity [25]. Recently, our team also found that suppression of Fyn alleviated the malignant phenotype of melanoma through downregulation of CD147 phosphorylation [21].

Considering the essential role of Fyn in various tumors, accumulating evidence demonstrated that Fyn is a novel potential target in antitumor drug treatment [26]. In our study, we used virtual screening based on ATP binding sites from our in-house library, and identified a novel compound (Lj-1-60) that targeted Fyn. Then, we confirmed that this compound directly binds to Fyn in vitro and in vivo (Fig. 2a, b). As is reported that PP1 and PP2 were developed as first generation SFK-selective tyrosine kinase inhibitors [27]. Then, a variety of SFK inhibitors have been investigated. Dasatinib, an SRC/ABL small molecular kinase inhibitor, has been approved by FDA due to the treatment of hepatocellular carcinoma [28], moreover, dasatinib inhibits cell proliferation in human esophageal squamous cell carcinoma through upregulating of the expression of MAD2 [29]. Masitinib, another SFK tyrosine kinase inhibitor, was assessed in a randomized controlled open-label trail, and showed that masitinib generated a statistically significant survival benefit over standard of care in advanced gastrointestinal stromal tumor [30]. Saracatinib (AZD0530) targets Fyn and inhibits the growth of lung cancer cells that are resistant to ALK inhibitors [31], interestingly, this compound also suppresses Fyn induced invasion and metastasis by decreasing Vimentin and Snail [32]. However, subsequent studies showed that saracatinib inhibits other SFKs kinase activities, such as Src, Yes, and Lyn. Stat3 contains 24 exons located within chromosome 17q21, which is highly conserved across species. Stat3 is one of the seven members of the STAT family continuously activated in various tumors including melanoma, and executes an essential role in multiple biological processes, such as cell proliferation, differentiation and immune responses [33]. The SH2 domain of Stat3 contains a serine phosphorylation site (S727) and a tyrosine phosphorylation site (Y705), phosphorylation of these sites of Stat3 were discovered to result in the formation of Stat3 homo- or heterodimers by interacting with SH2 domain [34]. It was reported that growth factor receptors or kinases such as epidermal growth factor receptor (EGFR), Janus Associated Kinases (JAK) and Src family members activate Stat3 [35]. It was shown that the continuously activation of Stat3 related with the aberrant activation of JAK and c-SRC in breast cancer cells, and inhibition of Src damaged the activity of Stat3 DNA-binding [36]. Additionally, Grandis et al. confirmed that c-Src interacts with Stat3 and mediates the activation of Stat3 [13]. Src family kinases including Src, Hck, Lyn, Fyn, and Fgr were reported to interact with stat3 [37]. Fyn, as a member of the Src family, has been well thoroughly studied. Stat3 is a well-known and broadly reported substrate of Fyn. Fyn deficiency is associated with attenuation of renal fibrosis through inhibition of p-Stat3 (Tyr 705) [38]. Further experiments shown that miR-125a-3p inhibits Fyn expression causing the inactivation of Fyn downstream effector Stat3 and attenuating pulmonary fibrosis [39]. In accordance with above these findings, we discovered through an in vitro kinase assay that Fyn phosphorylates Stat3 at tyrosine site 705, and Lj-1-60 inhibits the phosphorylation of Stat3 (Fig. 2c). In addition, our findings revealed that knocked down of Fyn decreased the phosphorylation of Stat3 in melanoma cells (Fig. 2d). Lj-1-60 treatment was also observed to inhibit the phosphorylation of Stat3 (Fig. 2e), indicating that Lj-1-60 might be a novel inhibitor of Fyn.

The effect of Lj-1-60 in melanoma cells was further investigated. Lj-1-60 significantly inhibited cell proliferation of melanoma cells, such as Sk-Mel-5, Sk-Mel-28 and A375, but it exhibited lesser cytotoxicity toward non-tumor cell such as PIG1, HaCaT, JB6 and H9C2 (Fig. 3, Additional file 4: Fig S3). Moreover, Lj-1-60 induced cell cycle arrest at the G2/M phase, and increased p-P53, P53, P21, Bax and cleaved PARP expression and downregulated Bcl-2 expression (Fig. 4), which is consistent with the inhibitory effect of Fyn expression on melanoma cells. The antitumor role of Lj-1-60 in melanoma was also confirmed in xenograft study in vivo (Fig. 5). Research on the effect of various inhibitors on melanoma has been widely documented. 3PO reported as a selective inhibitor of 6-phosphofructo-2-kinase/fructose-2,6-biphosphatase 3 (PFKFB3) has proapoptotic and anti-proliferative role in human melanoma cells A375 with BARF V600E mutation [40]. Juglone isolated from walnut trees synergizing BRAF inhibitor induced apoptosis in resistance melanoma cells A375R and SK-MEL-5R through ROS and p38-p53 pathway [41], while the effect of Lj-1-60 on resistance melanoma cells in our study needed to be further explored. A novel chalcone derivative reported by our lab, compared with Lj-1-60, has no specific target but exhibited inhibition of proliferation and induction of cell cycle arrest and apoptosis in melanoma through upregulating ROS signaling [16]. To investigate the potential mechanism of Lj-1-60 on melanoma, RNA-seq was conducted, and the results indicated that the cell cycle, apoptosis and DNA replication pathways were significantly altered after Lj-1-60 treatment (Additional file 5: Fig S4). We then validated the transcriptional expression of key candidate genes such as CDKN1A (P21), GADD45A, and MCM3 after this compound treatment and knocking down Fyn expression in melanoma cells (Fig. 6a, b). These candidate genes are crucial for cell cycle, apoptosis and DNA replication. P21 encoded by CDKN1A is a cyclin-dependent kinase (CDK) inhibitor (CKI), which is regulated by P53. The P21 expression is upregulated during various stimuli induced cell cycle arrest [42]. GADD45A is the most well known gene among the GADD family, and it is often related with growth inhibition and apoptosis. A well-known mechanism of GADD45A induction is that P53 binds to the third intron of the GADD45A by stimulating its transcription, and results in G2/M arrest [43]. The role of MCM3 is participating in DNA replication. It was reported that cleavage of MCM3 results in the inactivation of the MCM complex and prevents untimely DNA replication during the execution of cell death program [44]. Accumulating evidence had shown that the activation of Stat3 participates in oncogenesis through regulating cell cycle and apoptosis [45–47]. Chapman et al. reported that inhibition of Stat3 expression induces mammary epithelial apoptosis through up-regulating P53 and P21, indicating a key role for Stat3 in apoptosis [48]. Nakajima et al. [49] also discovered that blocking Stat3 activation led to cell growth arrest. Suppression of Stat3 expression induced Fas-mediated breast cancer cells apoptosis [50]. Mechanistically, Stat3 as a transcription factor has been found to control multiple downstream target genes such as cyclin D, C-Myc, Bcl-2, Bcl-xl, Mcl-1 and BIRC5 which is responsible for survival because it binds to promoter regions through recognizing the consensus site TTN_5_AA [51]. Research reported that interruption of IL-6 mediated Stat3 signaling leads to inhibition of Bcl-xl, which correlates with induction of apoptosis, and ectopic overexpression of Bcl-xl protects cells from apoptosis providing evidence that Bcl-xl executes the anti-apoptotic role in Stat3 pathway [52]. In addition to regulating the transcription of cell cycle and apoptosis related target genes, Stat3 activation in melanoma cells controls matrix metalloproteinase-2 (MMP-2) expression through interacting with MMP-2 promoter, thus promoting melanoma invasion and metastasis [53]. In our study, we observed that both Fyn deficiency and Lj-1-60 treatment induced cell cycle arrest and apoptosis in melanoma cells. Similarly, we also observed a decreasing level of Bcl-2 in Fyn deficiency and Lj-1-60 treated melanoma cells, which may explain why inactivated Stat3 cannot translocate into the nucleus and bind to the Bcl-2 promoter, thus decreasing the transcription of Bcl-2.

## Conclusion

In summary, we concluded that Lj-1-60 inhibited melanoma proliferation and induced cell cycle arrest into the G2/M phase and apoptosis by targeting Fyn/Stat3 pathway (Fig. 6c), indicating Lj-1-60 is a potential therapeutic chemical that could be used in the treatment of melanoma.

## Supplementary information

**Additional file 1: Table S1.** The primers used in the RT-PCR reaction.

**Additional file 2: Fig S1.** Bioinformatics analysis of the expression and OS of Fyn. (A) Scatter plots depict Fyn expression in GEO database (GSE114445, GSE29359). Nevus Melanocyte & primary melanoma; Melanocyte & melanoma.

**Additional file 3: Fig S2.** Screening out for Lj-1-60. (A) Cell viability of eighty-three chemicals screened out from our in-house library was detected. Melanoma cells Sk-Mel-28 were treated with a dose of 10 μM candidate chemicals for 48 h. (B) The chemical structural of Lj-1-60. (C, D) The sketch map of Lj-1-60 screened out through virtual molecular modeling interacts with Fyn kinase at residues of Met85, Asp148.

**Additional file 4: Fig S3.** Cell viability of tumor and non-tumor cells. (A) Cell viability of melanoma cell A375 treated with Lj-1-60 with indicated concentration. (B, C) Cell viability of HaCAT and JB6 was detected. Data were expressed as mean (n = 3) ± SD, **P < 0.01, ****P < 0.0001.

**Additional file 5: Fig S4.** Transcriptome analysis of melanoma cells treated with 2 μM Lj-1-60. (A, D) Clustering analyses of the effect of Lj-1-60 on the gene expression profile in melanoma cells Sk-Mel-5 (top) and Sk-Mel-28 (down). (B, E) KEGG pathway analyzed and the bubble chart indicated that the top 20 differential signaling pathways enriched in the Lj-1-60 treated melanoma cells Sk-Mel-5 (top) and Sk-Mel-28 (down). The x-axis represents the enrichment score, and the y-axis is the enriched pathways. (C, F) Gene set enrichment analysis (GSEA) revealed significant pathways associated with cell cycle phase transition signature (top) and DNA replication(down).

## Data Availability

RNA-seq data of this study was uploaded on NCBI (PRJNA634157).
