# Supplementary figures and images for: A novel chalcone derivative suppresses melanoma cell growth through targeting Fyn/Stat3 pathway
Source: Cancer Cell Int. 2020 Jun 18;20:256. doi: 10.1186/s12935-020-01336-2 (PMC7302361; doi:10.1186/s12935-020-01336-2)

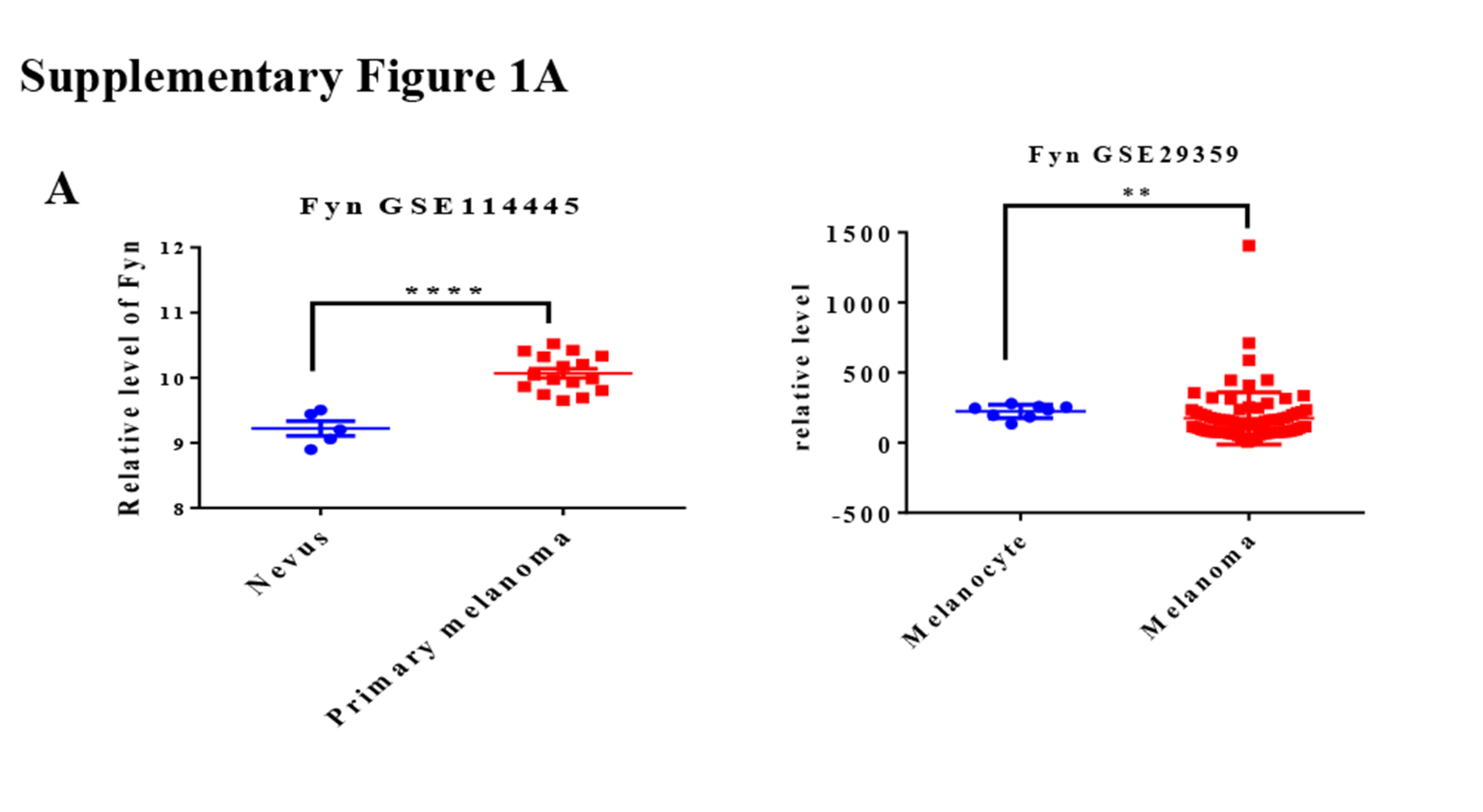

Supplement: Supplementary file 2 — Additional file 2: Fig S1. Bioinformatics analysis of the expression and OS of Fyn. (A) Scatter plots depict Fyn expression in GEO database (GSE114445, GSE29359). Nevus Melanocyte & primary melanoma; Melanocyte & melanoma. [file 12935_2020_1336_MOESM2_ESM.tif]

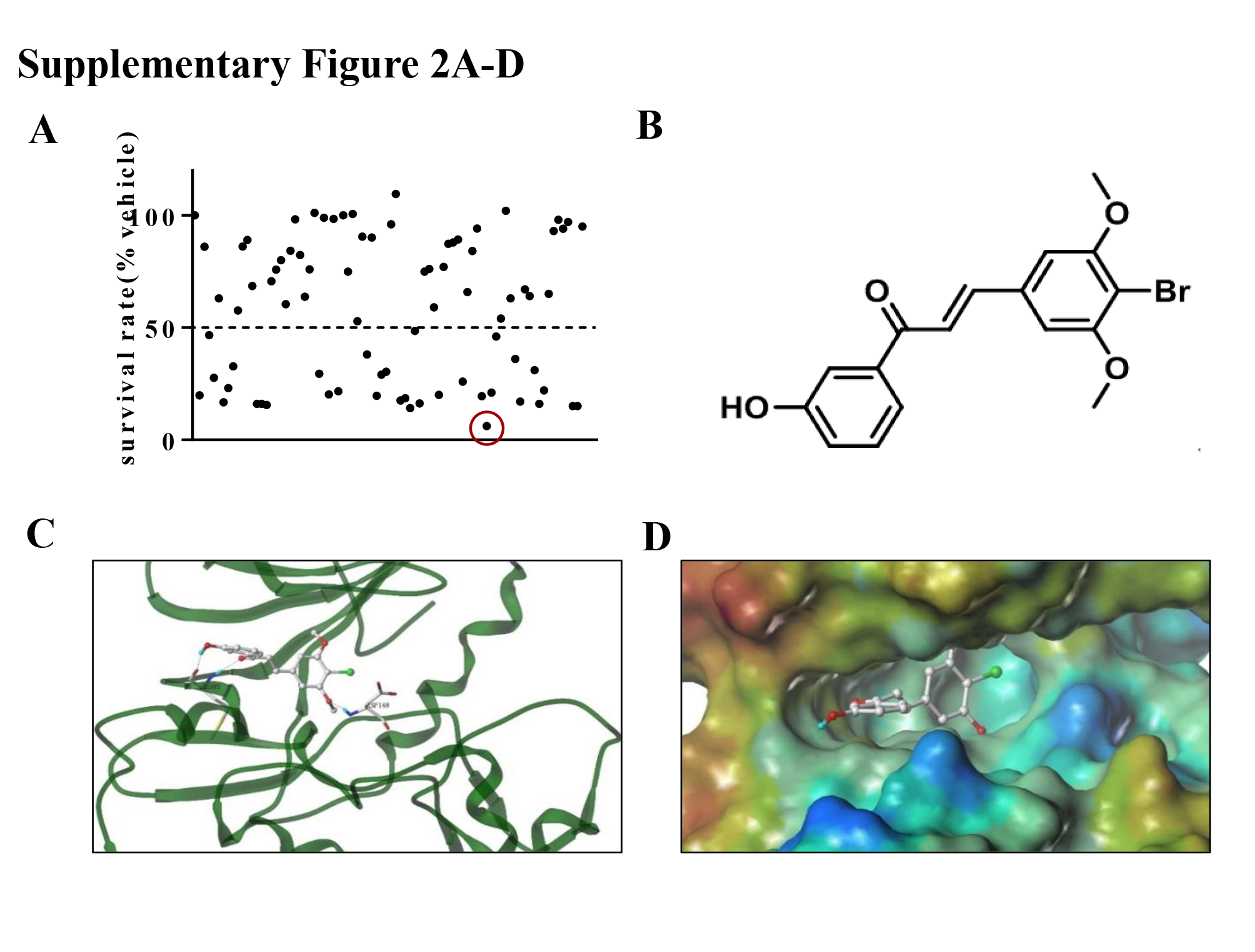

Supplement: Supplementary file 3 — Additional file 3: Fig S2. Screening out for Lj-1-60. (A) Cell viability of eighty-three chemicals screened out from our in-house library was detected. Melanoma cells Sk-Mel-28 were treated with a dose of 10 μM candidate chemicals for 48 h. (B) The chemical structural of Lj-1-60. (C, D) The sketch map of Lj-1-60 screened out through virtual molecular modeling interacts with Fyn kinase at residues of Met85, Asp148. [file 12935_2020_1336_MOESM3_ESM.tif]

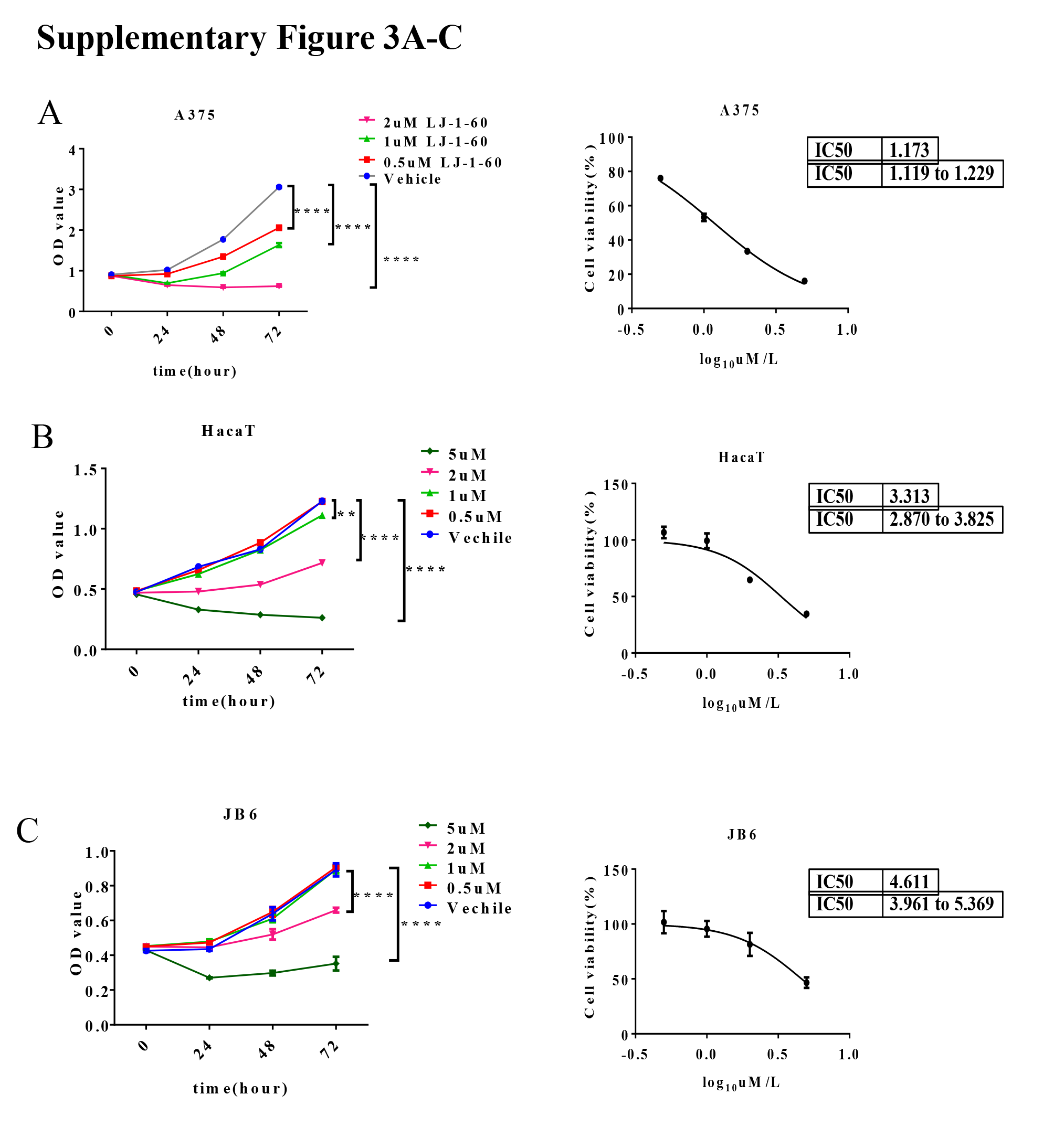

Supplement: Supplementary file 4 — Additional file 4: Fig S3. Cell viability of tumor and non-tumor cells. (A) Cell viability of melanoma cell A375 treated with Lj-1-60 with indicated concentration. (B, C) Cell viability of HaCAT and JB6 was detected. Data were expressed as mean (n = 3) ± SD, **P < 0.01, ****P < 0.0001. [file 12935_2020_1336_MOESM4_ESM.tif]

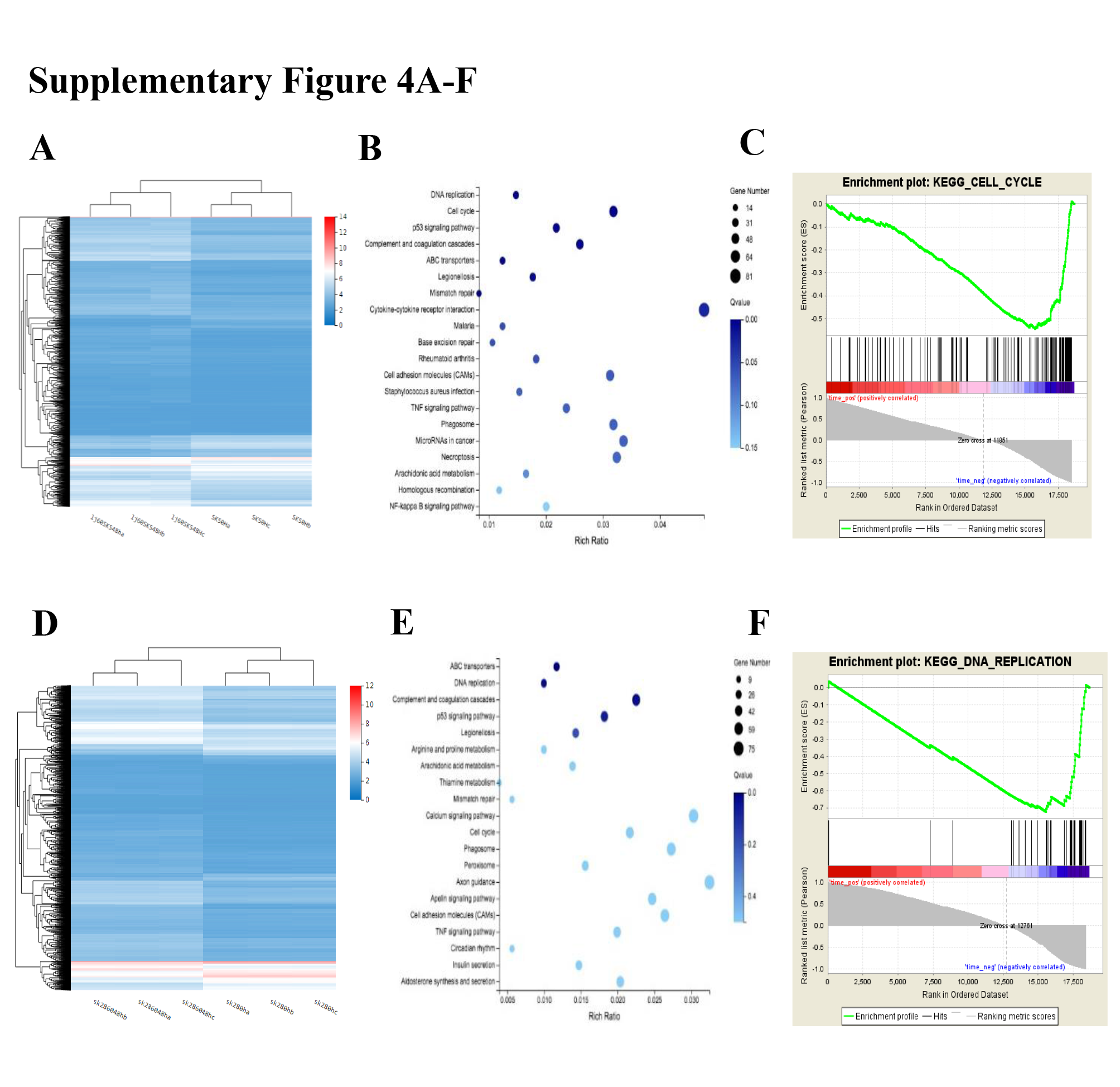

Supplement: Supplementary file 5 — Additional file 5: Fig S4. Transcriptome analysis of melanoma cells treated with 2 μM Lj-1-60. (A, D) Clustering analyses of the effect of Lj-1-60 on the gene expression profile in melanoma cells Sk-Mel-5 (top) and Sk-Mel-28 (down). (B, E) KEGG pathway analyzed and the bubble chart indicated that the top 20 differential signaling pathways enriched in the Lj-1-60 treated melanoma cells Sk-Mel-5 (top) and Sk-Mel-28 (down). The x-axis represents the enrichment score, and the y-axis is the enriched pathways. (C, F) Gene set enrichment analysis (GSEA) revealed significant pathways associated with cell cycle phase transition signature (top) and DNA replication(down). [file 12935_2020_1336_MOESM5_ESM.tif]
